# New Generation of Hybrid Materials Based on Gelatin and Bioactive Glass Particles for Bone Tissue Regeneration

**DOI:** 10.3390/biom11030444

**Published:** 2021-03-17

**Authors:** Amel Houaoui, Agata Szczodra, Mari Lallukka, Lamia El-Guermah, Remy Agniel, Emmanuel Pauthe, Jonathan Massera, Michel Boissiere

**Affiliations:** 1 Biomaterials for Health Research Group, ERRMECe, Equipe de Recherche sur les Relations Matrice Extracellulaire-Cellules (EA1391), Institut des Matériaux I-MAT (FD4122), CY Tech, CY Cergy Paris Université, Maison Internationale de la Recherche (MIR), rue Descartes, 95001 Neuville sur Oise CEDEX, France; amel.houaoui@cyu.fr (A.H.); lamia.el-guermah@cyu.fr (L.E.-G.); remy.agniel@cyu.fr (R.A.); emmanuel.pauthe@cyu.fr (E.P.); 2 Tampere University, Faculty of Medicine and Health Technology, Laboratory of Biomaterials and Tissue Engineering, Korkeakoulunkatu 3, 33720 Tampere, Finland; agata.szczodra@tuni.fi (A.S.); mari.lallukka@polito.it (M.L.); jonathan.massera@tuni.fi (J.M.)

**Keywords:** hybrid scaffold, bioactive glass, gelatin, GPTMS, bone tissue engineering

## Abstract

Hybrid scaffolds based on bioactive glass (BAG) particles (<38 µm), covalently linked to gelatin (G*) using 3-glycidoxypropyltrimethoxysilane (GPTMS), have been studied for bone bioengineering. In this study, two glass compositions (13-93 and 13-93B20 (where 20% of the SiO_2_ was replaced with B_2_O_3_)) were introduced in the gelatin matrix. The C_factor_ (gelatin/GPTMS molar ratio) was kept constant at 500. The hybrids obtained were found to be stable at 37 °C in solution, the condition in which pure gelatin is liquid. All hybrids were characterized by in vitro dissolution in Tris(hydroxymethyl)aminomethane (TRIS) solution (for up to 4 weeks) and Simulated Body Fluid (SBF) (for up to 2 weeks). Samples processed with 13-93B20 exhibited faster initial dissolution and significantly faster precipitation of a hydroxyapatite (HA) layer. The faster ion release and HA precipitation recorded from the G*/13-93B20 samples are attributable to the higher reactivity of borosilicate compared to silicate glass. The MC3T3-E1 cell behavior in direct contact with the hybrids was investigated, showing that the cells were able to proliferate and spread on the developed biomaterials. Tailoring the glass composition allows us to better control the material’s dissolution, biodegradability, and bioactivity. Bioactive (especially with 13-93B20 BAG) and biocompatible, the hybrids are promising for bone application.

## 1. Introduction

Bone fractures are a common trauma. For a large loss of bone substance (defect greater than 1 cm^3^) following a traumatic situation as a pathology or accidental defect, the natural process of self-repair is compromised [1]. Tissue engineering is an innovative approach used for bone repair. Bone reconstruction is assisted with materials that participate in tissue regeneration [2,3]. These materials must have properties adapted to this function. 

Bioactive ceramics are of interest in bone regeneration. The bioactivity of these materials results in the appearance of biological activity in the host organism and the existence of ion exchanges between the material and living tissue [4]. Synthetic hydroxyapatite (HA) and β-tricalcium phosphate (β-TCP) are more widely used [5,6,7]. They often have osteoconductive and sometimes osteoinductive properties. However, their slow resorption limits their clinical relevance [8,9]. Indeed, the limited resorption of those ceramics was demonstrated in-vivo when used in cements [10].

Bioactive Glass (BAG) is a subcategory of ceramics and are not only osteoconductive but also osteoinductive [11]. These glasses are mainly used for hard tissue reconstruction, but they are also able to bond to soft tissue [12]. However, due to their brittleness, shaping the glass into its final shape remains a challenge. Organic/inorganic composite scaffolds represent a convenient alternative to traditional BAGs. They allow for the possibility to tailor the mechanical properties, degradation kinetics, and bioactivity. Current research is focused on the fabrication of bioactive composite materials, with the bioactive phase incorporated as a filler into the bioresorbable polymer matrix [13]. However, a drawback with many conventional composites is that the constituent phases interact on a micrometer scale, which can result in differential resorption rates during dissolution and masking of the bioactive component. This would inevitably lead to material instability in vivo [14]. 

Hybrid scaffolds are materials where the organic and inorganic phases interact chemically on a nanoscale, allowing us to control the properties of the final material, for instance, degradation rates and mechanical properties [14,15,16]. Moreover, the inorganic and organic contents in addition to the degree of covalent links can be adjusted for precise control of the hybrid properties as stiffness and dissolution rates, leading to material adapted for a specific application [14]. For hybrid formation, covalent links between organic and inorganic matrixes are fundamental. They can be obtained through reaction with organosilanes such as 3-glycidoxypropyltrimethoxysilane (GPTMS) or 3-aminopropyltriethoxysilane (APTES) [17,18,19,20].

**Figure 1:**
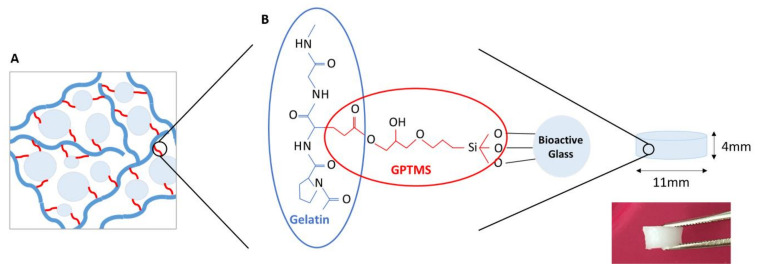
**(A**) Schematic representation (inspired from Mahony et al. [18]) and (**B**) chemical structure of the hybrid made of gelatin and bioactive glass (BAG) covalently linked by 3-glycidoxypropyltrimethoxysilane (GPTMS).

In our work, we present hybrid materials for bone tissue engineering based on gelatin and BAG (Figure 1), where two BAGs, with different compositions (13-93 and 13-93B20), are compared. 13-93 is an Food and Drug Administration (FDA)-approved BAG that exhibits a slower dissolution rate than commercialized BAGs 45S5 or S53P4 [21,22]. The rational for studying 13-93 BAG lies in preventing excessive dissolution of the glass particles during processing, degradation of the organic phase, a decrease in the mechanical properties, and a fast release of ions that could be toxic for the cells. 13-93B20, a similar composition to 13-93 but with 20% of the SiO_2_ substituted with B_2_O_3_, was also included in the study. It has been showed that the borosilicate glasses based on the 13-93 composition have faster in vitro dissolution but also faster conversion to HA while maintaining a slower dissolution kinetics than 45S5 and S53P4 [13,23,24]. 

Here, two hybrid materials were studied, based on gelatin and 13-93 or 13-93B20. The content of the organic and inorganic matrixes was 70/30 (wt%), and a C_factor_ (degree of covalent coupling, molar ratio of GPTMS/gelatin) of 500 was used. To investigate their in-vitro dissolution, the hybrids were immersed in Tris(hydroxymethyl)aminomethane (TRIS) buffer solution for one month. Ion release from the glass was quantified, and the mineral content was measured. The compressive properties of the hybrids were studied during the immersion. The bioactivity, assumed to be related to the precipitation of a HA layer at the materials’ surface when immersed in aqueous solution, was assessed in Simulated Body Fluid (SBF) [25]. Preliminary cell experiments were performed to assess cell activity by culturing MC3T3-E1 pre-osteoblastic cells at the surface of the hybrids. Cell proliferation and morphology were studied. The aim of this study was to assess the stability of the hybrids, in vitro, in aqueous solutions and its biocompatibility. 

## 2. Materials and Methods

### 2.1. Materials Preparation and Characterization

#### 2.1.1. Bioactive Glass Processing

BAGs 13-93 and 13-93B20 were prepared from analytical grade K_2_CO_3_ (Alfa Aesar, Ward Hill, MA, USA), (Na_2_CO_3_, NH_4_H_2_PO_4_, (CaHPO_4_)(2(H_2_O)), CaCO_3_, MgO, H_3_BO_3_ (Sigma Aldrich, St Louis, MO, USA), and Belgian quartz sand. The 100 g batches of 13-93 and 13-93B20 were melted for 3 h at 1450 °C in a platinum crucible. The molten glasses were cast, annealed, crushed, and finally sieved into less than 38 µm particles. The glasses were dried at 200 °C for 2 h prior to be used. The nominal oxide compositions of the glasses are given in Table 1. 

#### 2.1.2. Hybrids Synthesis

Gelatin (Porcine, Type A, Bloom 300, Sigma Aldrich, St Louis, MO, USA) was dissolved at 37 °C in 10 mM hydrochloric acid (HCl, Merck Millipore, Burlington, MA, USA) at a concentration of 50 mg·mL^−1^. This solution was functionalized by adding GPTMS (Sigma Aldrich, St Louis, MO, USA) to obtain a C_factor_ (molar ratio of GPTMS/gelatin) of 500. Different C_factor_ have been studied (data not shown), and this parameter was optimized in order to limit the cellular toxicity of GPTMS. The functionalized gelatin (G*) solution was mixed 2 h at 37 °C. The 13-93 or 13-93B20 BAG particles were added and mixed for 1 h at 37 °C for a ratio of G*/BAG of 70/30 wt%. This ratio was optimized (data not shown) to obtain enough covalent links in the structure to obtain a gel self-supported at biological temperature. Finally, sodium fluoride 1% (NaF, Sigma Aldrich, St Louis, MO, USA) was added to catalyze the inorganic condensation reaction. The final solution was mixed for 10 min at 37 °C. The solution was poured in silicon molds and left to gel at room temperature for 24 h. 

Hybrid materials with a diameter of 11 mm and height of 4 mm were cut. To measure their glass content, they were freeze-dried and heated for 2 h at 500 °C under air to remove all the organic phase. The remaining mineral phase was weighed. The measure was repeated on 4 samples per composition, and the average glass content with standard deviation was calculated.

### 2.2. Behavior of G*/BAG Hybrids

#### 2.2.1. Physicochemical Properties of the Hybrids

##### Immersion in TRIS

Tris(hydroxymethyl)aminomethane (TRIS) solution (50 mM) was prepared by mixing ultrapure TRIS (Sigma Aldrich, St Louis, MO, USA) and TRIS-HCl (Sigma Aldrich, St Louis, MO, USA) in ultrapure water, and the pH was adjusted to 7.4 at 37 °C. The samples were punched to obtain small cylinders of 11 mm diameter and 4 mm height (≈380 mg), which were immersed in 30 mL of TRIS solution for up to 28 days at 37 °C on an agitator (Heidolph Instruments, Schwabach, Germany) with an orbital speed of 100 rpm. To avoid saturation of the solution with the ions released from the hybrids, the TRIS buffer was refreshed each week. 

At the desired time of immersion, the concentration of elements released from the hybrids was studied by diluting 500 µL of the immersion solution in 4.5 mL of ultrapure water for ion analysis. Inductively Coupled Plasma - Optical Emission Spectroscopy (ICP-OES) (Agilent technologies 5110, Santa Clara, CA, USA) was employed to quantify P (λ = 253.561 nm), Ca (λ = 422.673 nm), Mg (λ = 279.553 nm), Si (λ = 250.690 nm), B (λ = 249.678 nm), K (λ = 766.491 nm), and Na (λ = 589.592 nm) concentrations in the solution after samples immersion. The measurements were conducted in four separate samples at each time points for each composition, and the results are presented as mean ± standard deviation (SD). 

##### Mineral Content in Hybrids

Mineral mass after the samples’ synthesis and at various immersion times was measured after freeze-drying and burning the samples for 2 h at 500 °C under air to remove all the organic phase. The remaining mineral phase was weighed. The measurements were conducted on four separate samples at each time points for each composition, and the results were presented as mean ± SD.

##### Mechanical Properties of the Hybrids

The mechanical properties of the hybrids after synthesis and after immersion (wet) were tested by compression test at room temperature using a texturometer (LS1, Lloyd Instruments, Ametek, Berwyn, PA, USA). The measurements were conducted on four separate samples at each time point for each composition. A 20 N load cell was used for testing, with a compression extension speed of 1 mm.min^−1^. The results are presented as mean ± SD. 

#### 2.2.2. Hybrids Bioactivity

##### Immersion in Simulated Body Fluid (SBF)

Developed by Kokubo et al., SBF was prepared following the methodology from the standard ISO/FDIS 23317 [26]. The samples were punched to obtain small cylinders of 11 mm diameter and 4 mm height (≃380 mg), which were immersed in 30 mL of SBF for up to 2 weeks at 37 °C on an agitator (Heidolph Instruments, Schwabach, Germany) with an orbital speed of 100 rpm. During the experiment, the solution was not refreshed to study the precipitation of calcium phosphate. The ion concentration in the solution according to immersion time was measured as previously described. The measurements were conducted in four separate samples at each time points for each composition, and the results are presented as mean ± SD.

##### Mineral Content in Hybrids

Mineral mass after various immersion times was measured as explained above. Measurements were conducted on four separate samples at each time point for each composition, and the results are presented as mean ± SD.

##### Hybrids Surface Analysis

The reactive layer on the hybrid surface after immersion in SBF was observed by SEM (GEMINISEM 300 from Zeiss, Iena, Germany), and its composition was analyzed by Energy-Dispersive X-ray spectroscopy (EDX Quantax from Bruker, Billerica, MA, USA). The Infrared (IR) absorption spectra of the hybrids immersed in SBF were also recorded using a Bruker Alpha FTIR in Attenuated Total Reflectance (ATR) mode. The measurements were performed on dry samples. All IR spectra were recorded within the range 399–4000 cm^−1^ with a resolution of 2 cm^−1^ and 32 accumulation scans.

#### 2.2.3. Cell Analysis

##### Hybrids Preparation

G*/13-93 and G*/13-93B20 hybrids were synthesized in sterile conditions. The samples were punched to obtain small cylinders of 11 mm diameter and 4 mm height. Each material of each condition was immersed in sterile TRIS solution during 10 days at 37 °C to remove excess components. After that, all cell experiments were performed in 48-well plates.

##### Cell Culture

Pre-osteoblastic MC3T3-E1 cells subclone four (ATCC, Manassas, VA, USA) were cultured in α-Minimum Essential Media (α-MEM) (Gibco, Life Technologies, Carlsbad, CA, USA) containing glutamine supplemented with 10% Fetal Bovine Serum (FBS, Biosera, Marikina, Philippines) and 1% penicillin/streptomycin (Gibco, Life Technologies, Carlsbad, CA, USA). The cells were cultured with a humidified atmosphere of 5% CO_2_ at 37 °C.

##### Cell Proliferation 

To compare the behavior of MC3T3-E1 cells on the different samples, cell proliferation was studied using a CyQUANT Cell Proliferation Assay kit (Invitrogen, Life Technologies, Carlsbad, CA, USA). The control used was the Tissue Culture Polystyrene (TCPS) 48 well-plate. The hybrids were placed in the wells, and 5000 cells/well were seeded. The medium was changed every 2 days. After 1, 3, 7, 10, and 14 days of culture, the cells were lysed with 700 µL 0.1% Triton-X100 (Fisher Scientific, Hampton, NH, USA) buffer and conserved at −80 °C. After one freeze–thaw cycle, three 20 μL aliquots of each lysate were pipetted to a black 96-well plate (Corning, Corning, NY, USA) and mixed with 180 μL working solution containing CyQUANT GR dye and cell lysis buffer. The fluorescence at 520 nm was measured with a Spectrofluorometer Xenius XM (Safas, Monaco). 

##### Cell Morphology

The morphology of the cells on the different samples was observed after 1, 7, and 14 days of culture. The control used was TCPS coverslips (Nunc Thermanox Coverslips, ThermoFisher Scientific, Waltham, MA, USA) of 13 mm diameter in a 24-well plate. The number of cells was adjusted according to the well area. The same density of cells was seeded on the hybrids and the TCPS coverslips controls, and after each time point, the cells were fixed with 4% (*w*/*v*) para-formaldehyde solution for 15 min and then permeabilized with 0.1% (*v*/*v*) Triton X-100 (Sigma Aldrich, St Louis, MO, USA) for 10 min. Nonspecific binding sites were blocked by incubating the disks in Phosphate Buffered Saline (PBS) containing 1% Bovine Serum Albumin (BSA, Sigma Aldrich, St Louis, MO, USA) for 1 h. The cytoskeleton and nuclei of the cells were stained, respectively, with 1:500 diluted TRITC-labelled phalloidin (Sigma Aldrich, St Louis, MO, USA P1951) and 1:1000 diluted 4′,6-Diamidino-2-phenylindole dihydrochloride (DAPI, Sigma Aldrich, St Louis, MO, USA D9542) in PBS–BSA 0.5% for 1 h. Each incubation with antibodies was performed in wet and dark conditions. The samples were then washed in PBS–BSA 0.5% and pure water and observed using a LSM710 confocal microscope (Zeiss, Iena, Germany).

##### Statistical Analysis

Data were analyzed using GraphPad Prism 8 Software. Statistical significance between groups was assessed by one-way analysis of variance (ANOVA). The experimental results are expressed as means ± standard deviation. Statistical significance is taken for values of *p* < 0.01.

## 3. Results and Discussion

The aim of this study is to develop hybrid materials based on gelatin and BAG able to release therapeutic ions for bone regeneration while controlling the dissolution properties of the organic and inorganic phases of the scaffold. The first step was to determine the optimal C_factor_ for targeted applications. Indeed, the higher the C_factor_, the more covalent links will be formed in the structure. However, an excess of GPTMS can lead to excessive unreacted precursor, leading to cellular toxicity [14,27]. Thus, to avoid this negative effect, studies have led us to use a C_factor_ of 500 for our hybrids (data not shown). Therefore, the BAGs 13-93 and 13-93B20 were covalently linked to gelatin with a C_factor_ of 500. The materials dissolution in aqueous solutions and their bioactivity were compared.

### 3.1. Characterization of the Hybrids after Synthesis

Table 2 presents the glass loading in the hybrids and their young modulus after synthesis. The mineral mass in the hybrids was determined after freeze-drying and burning the inorganic phase at 500 °C under air for 2 h. Table 2 shows that the glass loading is 34 ± 2 wt% and 33 ± 1 wt% for G*/13-93 and G*/13-93B20, respectively, as expected from the targeted loading.

Young’s modulus of G* alone and the hybrids were measured by a compression test. Both hybrids have Young’s moduli stable in wet and dry conditions. Young’s modulus of the hybrids is lower compared to that for G* alone. This shows that adding the glass in the organic phase influences the mechanical behavior of the scaffolds. This decrease occurs with both BAGs, showing that this is not due to the type of glass. It is interesting to note that, in the case of composite materials, the addition of mineral particles reinforces the mechanical properties [8,9]. However, for our hybrid materials, a decrease in Young’s modulus is observed probably because the glass induces defects on the gel structure and adds some heterogeneity. This heterogeneity in turn leads to weak points in the material, which become more friable. 

### 3.2. Behavior of the Hybrids in Solution

Resorbable materials need to present controlled degradation and sufficient mechanical properties until bone tissue regeneration [26]. Their bioactivity is a fundamental property that will help bone repair. To assess these properties, the hybrids were immersed in aqueous solutions and their dissolution was studied. 

#### 3.2.1. Dissolution in TRIS

**Figure 2:**
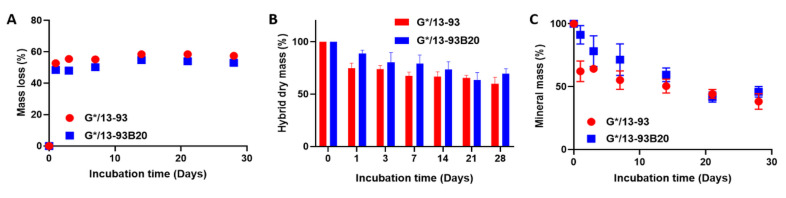
Mass loss (**A**), dry mass (**B**), and mineral mass (**C**) remaining in the hybrid G*/13-93 (●) and G*/13-93B20 (∎) as a function of immersion time in TRIS.

The hybrid degradation in TRIS was studied by mass measurements, ICP-OES analysis, and compression tests. These tests could not be done on G* without BAG because it was dissolving at 37 °C, contrary to G*/13-93 or 13-93B20. This shows that the materials made of gelatin and BAG, covalently linked by the GPTMS, can be considered as hybrids. Moreover, Mahony et al. worked on hybrids based on gelatin and silica network coming from Tetraethyl orthosilicate (TEOS) hydrolysis and condensation and showed that GPTMS is efficient in creating covalent links between both matrices [14]. Figure 2 presents the mass loss of the hybrids as a function of the immersion time (Figure 2A), the hybrid mass after freeze-drying (Figure 2B), and the mineral mass remaining in the materials after immersion (Figure 2C).

Mass loss graph (Figure 2A) shows that, after 24 h, the mass loss reaches 50% and remained stable at longer immersion times. The same results are observed for G*/13-93 and G*/13-93B20, showing that this mass loss is not dependent on the type of glass. In Figure 2B, a decrease in the hybrid dry mass is observable. At 24 h, the hybrid dry mass goes from 100% to 75% and 89% for G*/13-93 and G*/13-93B20, respectively, and does not reach 50% during the immersion. This result does not corroborate the mass loss at 24 h in Figure 2A. This means that, during the first 24 h, the hybrids lose mostly water. This can be seen from a macroscopic point of view on the hybrids that shrink and lose 2 mm of diameter and 1 mm of height after 24 h of immersion, indicating a rearrangement of the hybrids structure due to a syneresis phenomenon. 

**Figure 3:**
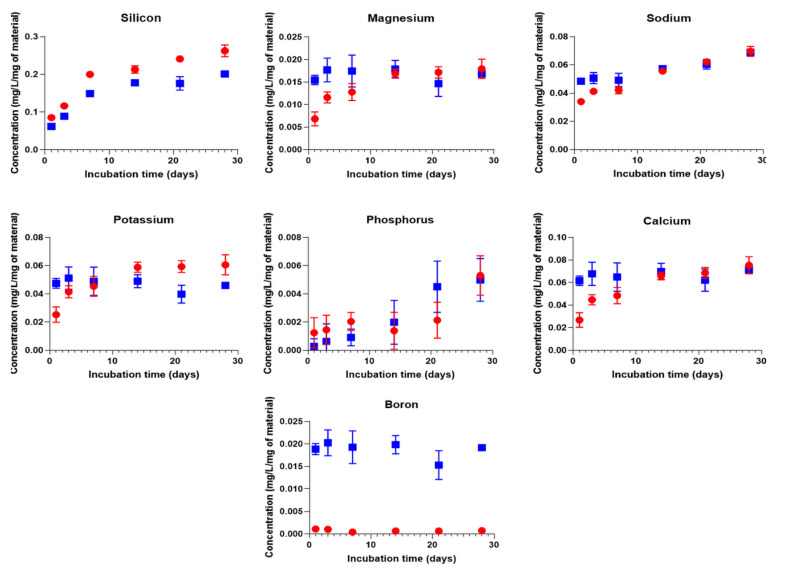
Elements concentrations of Si, Mg, Na, K, P, Ca, and B in the dissolution products of G*/13-93 (●) and G*/13-93B20 (∎) immersed in TRIS as a function of time. The concentrations are normalized to the sample mass.

In Figure 2B, the hybrid dry mass decreases with immersion time, exhibiting a dissolution of the material. In Figure 2C, the mineral mass decreases with immersion time. This result shows that 13-93 and 13-93B20 dissolve in TRIS solution during immersion. This is further confirmed by the quantification of ion release in solution (Figure 3).

**Figure 4:**
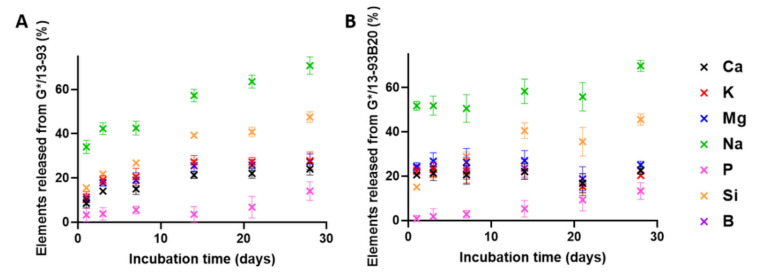
Release of ions from the (**A**) G*/13-93 and (**B**) G*/13-93B20 hybrids, immersed in TRIS as a function of time.

For both hybrids, all ions from the BAG are found to leach out into the solution, showing that the glasses dissolve through the gelatin. The Si release from G*/13-93B20 continuously increases and is slightly lower than that for G*/13-93. Moreover, the initial release of Mg, Na, K, and Ca elements is faster for G*/13-93B20 than for G*/13-93. This can be due to the fact that the borosilicate glass is more reactive with siloxane than G*/13-93, leading to a lower Si release from the hybrids with 13-93B20 than the one with 13-93. The release of the elements coming from G*/13-93B20 directly reaches the plateau from the beginning of the immersion, while it increases for G*/13-93 until it reaches the same plateau (Figure 3 and Figure 4). For G*/13-93 (Figure 4A), it appears that the glass dissolution is limited for Ca, K, and Mg, with a plateau reached at 14 days and approximately 20% of these elements released in solution. The Si and Na releases seem to be higher than the first ions cited, linear and continuous for 28 days. Figure 4B presents the release of ions from the hybrids containing 13-93B20 glass. For Ca, K, Mg, and B, the release of these elements is already at the plateau from the first time point contrary to the hybrid containing 13-93 (results also observed in Figure 3). This plateau, reached from the beginning of the immersion at 20% (as for G*/13-93), shows also that the initial dissolution of G*/13-93B20 is faster than for G*/13-93. 

For both hybrids, the Si and Na releases are more important than for the other elements, with a Na release already stable from the first time point for G*/13-93B20. The higher concentrations of Si and Na are probably because they come not only from the glass but also from the GPTMS and NaF, respectively. The important release of Si in solution is probably followed by condensation and polymerization, forming an amorphous silica-rich layer around the glass [28], slowing down the release of Ca, Mg, K, and B (for G*/13-93).

**Figure 5:**
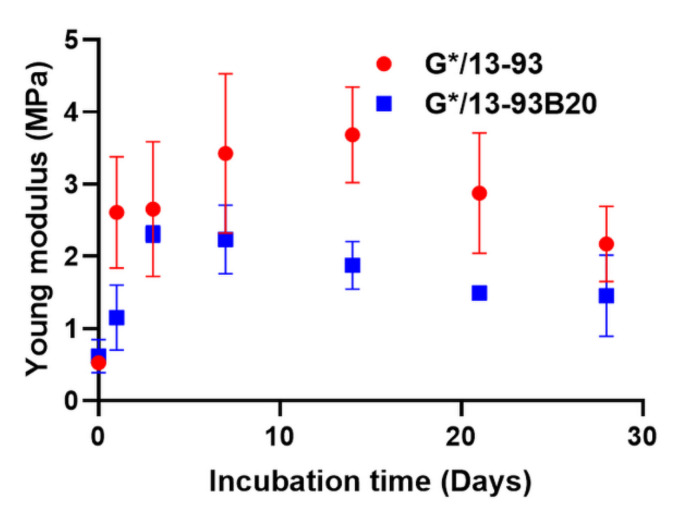
Young’s modulus measured by a compression test of wet G*/13-93 (●) and G*/13-93B20 (∎) hybrids as a function of immersion time in TRIS.

After analyzing degradation of the hybrids, their mechanical properties were studied with a compression test on wet samples during the immersion (Figure 5). 

Young’s modulus was measured on wet samples as a function of incubation time (Figure 5). The evolution of the mechanical properties takes place in two stages. First, an increase in Young modulus for both hybrids can be seen. A maximum is then reached at 3 days for G*/13-93B20 and at 14 days for G*/13-93. At longer immersion times, a decrease in Young’s modulus is noticed. The increase in modulus could be due to the syneresis phenomenon, as explained for Figure 2. The water loss leads to a reinforcement of the mechanical properties. After that, the decrease in Young’s modulus would be due to hybrids erosion, inducing a loss of its mechanical properties. This corroborates the phenomenon showed in Figure 3 and Figure 4, showing that this decrease happens when the stabilization of ion release is reached for G*/13-93.

Hybrids immersion in TRIS allowed to understand their dissolution and their ions release in solution. The 13-93B20 dissolves and reaches the saturation faster but finally at the same level than the 13-93. This dissolution has an influence on the mechanical properties but it should be noted that despite these variations, Young’s modulus stays close to that of cancellous bone [29,30,31].

#### 3.2.2. Dissolution in Simulated Body Fluid (SBF)

The hybrids were immersed in Simulated Body Fluid (SBF) to study their bioactivity. ICP-OES analysis, mass measurements, SEM observations, and EDX and FTIR analysis were conducted to assess the ion release/precipitation and the formation of a reactive layer.

**Figure 6:**
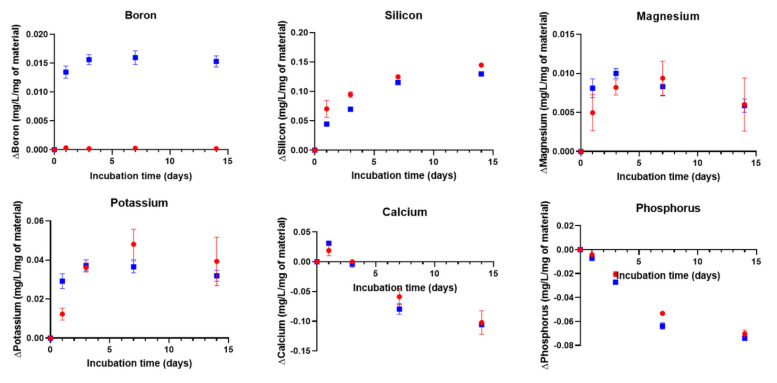
Concentrations of Si, Mg, K, P, Ca, and B in the dissolution products of G*/13-93 (●) and G*/13-93B20 (∎) immersed in Simulated Body Fluid (SBF) as a function of time. The concentrations are normalized to the sample mass. ΔElement = [Element] in SBF in the presence of the sample – [Element] in SBF initial solution.

As postulated by L.L. Hench, the ability of a material to induce precipitation of an hydroxyapatite layer at its surface can be considered as a sign of bioactivity [11]. Immersion in SBF was conducted for two weeks, and the solution was not refreshed. The ion concentration in the solution was quantified. The difference between the ion concentration in SBF and ion concentration after hybrid immersion was calculated (Figure 6).

The Ca concentration seems to increase initially and then decreases with immersion time, whereas the P concentration decreases from the beginning of the dissolution. This phenomenon was also observed in our previous study on composites based on Poly(Lactic) Acid (PLA) and the same glasses [13]. Generally, the decrease in Ca and P in SBF corresponds to the precipitation of a calcium-phosphate reactive layer. The elements Mg and K show similar trends during immersion in SBF. The 13-93B20 glass leaches out its ions at a faster rate initially than 13-93 and then; for both hybrids, a decrease in Mg and K concentrations appears. It is important to note that the dissolution rate slows down at earlier immersion times in SBF than in TRIS for G*/13-93. This decrease shows the saturation of the solution and probably that Mg and K can be incorporated into the calcium-phosphate reactive layer [32]. Silicon release is linear and continuous, tending towards a plateau, for both hybrids. It is initially higher for G*/13-93, which can be, as explained above, because the borosilicate glass would be more reactive with the siloxane, leading to a lower Si release from the hybrids with 13-93B20.

**Figure 7:**
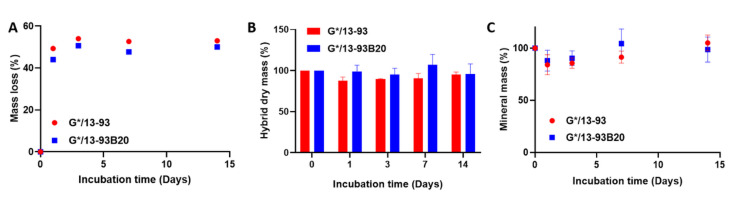
Mass loss (**A**), dry mass (**B**) and mineral mass (**C**) remained in the hybrid G*/13-93 (●) and G*/13-93B20 (∎) as a function of immersion time in SBF.

Figure 7 presents the mass loss of the hybrids as a function to immersion time in SBF (Figure 7A), the hybrid mass after freeze-drying (Figure 7B), and the mineral mass remaining in the materials after immersion (Figure 7C).

Figure 7A shows that, after 24 h, the mass loss reaches approximately 50%, corresponding to a water loss and thus a shrinking of the materials due to syneresis, as explained for TRIS immersion. Then, the mass loss stays stable during immersion. In Figure 7B, the dry mass of hybrids immersed in SBF does not show the same evolution as in TRIS immersion. Indeed, a decrease in the dry mass was observed in TRIS immersion, while in SBF, it appears to stay approximately stable. For the mineral mass (Figure 7C), while it decreases during immersion in TRIS, showing the dissolution of the glasses, in SBF, it decreases and then increases. This corresponds to dissolution of the glass followed by the precipitation of the calcium-phosphate layer. 

**Figure 8:**
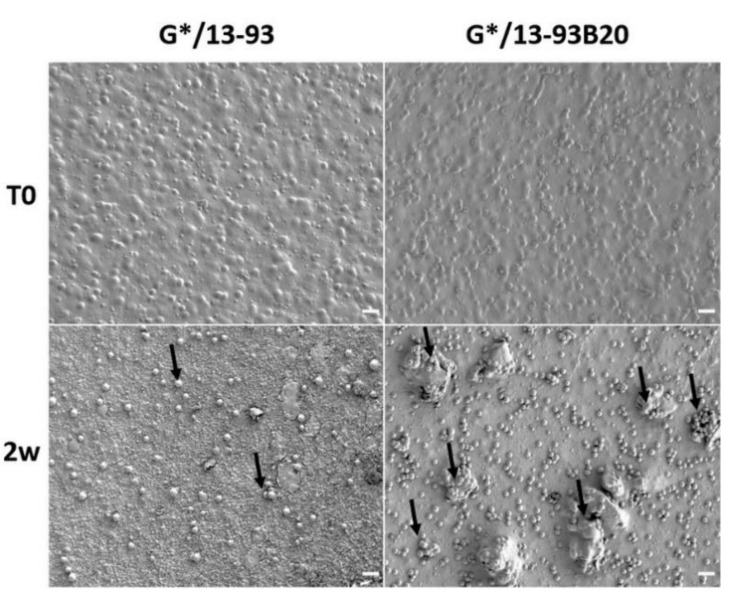
SEM image of the hybrid surface before and after 14 days of immersion in SBF (scale bar 20 μm).

**Figure 9:**
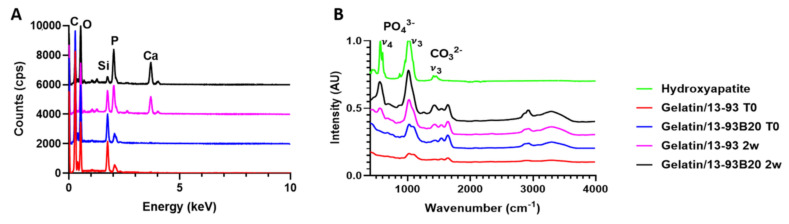
(**A**) EDX analysis of the nodules at the hybrids surface and (**B**) FTIR analysis of the samples surfaces before and after 14 days of immersion.

To assess the precipitation of this reactive layer, the hybrids were observed and analyzed using SEM/EDX as well as FTIR (Figure 8 and Figure 9).

After 2 weeks of immersion in SBF, nodules appeared at their surface. The nodules are small and dispersed on the hybrid containing 13-93, while they are more numerous and larger on the G*/13-93B20 hybrid surface. EDX analysis were performed on the nodules shown in Figure 8, and the spectra are presented in Figure 9A. The composition of the spheres from both hybrids is mainly Ca and P with a ratio of Ca/P of 1.77 ± 0.08, which is close to hydroxyapatite [33]. This corroborates the precipitation of the calcium-phosphate layer hypothesized from the ICP analysis (Figure 6) and confirms that these nodules are probably apatite nodules. It is interesting to point out that the materials containing the glass 13-93B20 exhibit a higher population and bigger nodules than materials processed with the glass 13-93. This is in agreement with Huang et al., who demonstrated that the borosilicate bioactive glasses convert to HA faster and more completely than their silicate counterpart [34].

The nature of the Ca/P precipitate was further analyzed by FTIR spectroscopy (Figure 9B). Two peaks at 500–600 cm^−1^ and ≈1000 cm^−1^ appear after G*/13-93B20 (they are also present for G*/13-93 but with a lower intensity) immersion in SBF. These peaks correspond to ν_4_ (P–O bending) and ν_3_ (P–O stretching) PO_4_^3−^ vibrations, respectively, in the apatite structure. The carbonate CO_3_^2−^ vibration is also present [35,36,37]. These peaks are characteristic of a hydroxyapatite structure. This is a good indication that the calcium phosphate layer precipitating on the hybrid surface is a hydroxy-carbonated apatite, indicative of the potential bioactivity of those materials. These results show the difference in reactivity between both glasses. Ion release is slowed down by the organic matrix barrier, but 13-93B20 allows us to remedy this effect compared to 13-93 glass. 

We developed gelatin/BAG hybrids using GPTMS as a coupling agent, with a C_factor_ of 500, using a sol-gel method. The target mineral content (70/30 wt%) was guaranteed by careful control of the processing steps. The mineral phase dissolves when immersed in aqueous solution with kinetics depending on the glass composition. The mechanical properties varied per the dissolution of the materials; however, Young’s modulus remained close to the value reported for cancellous bone [29,30,31]. Both hybrids were found to precipitate hydroxy-carbonated apatite during immersion in SBF. The bioactivity seemed significantly higher when using 13-93B20 glass. Therefore, preliminary cell experiments were conducted to assess if the hybrids are biocompatible and, thus, support the growth of pre-osteoblastic cells, which is fundamental for bone application.

### 3.3. MC3T3-E1 Proliferation and Morphology

**Figure 10:**
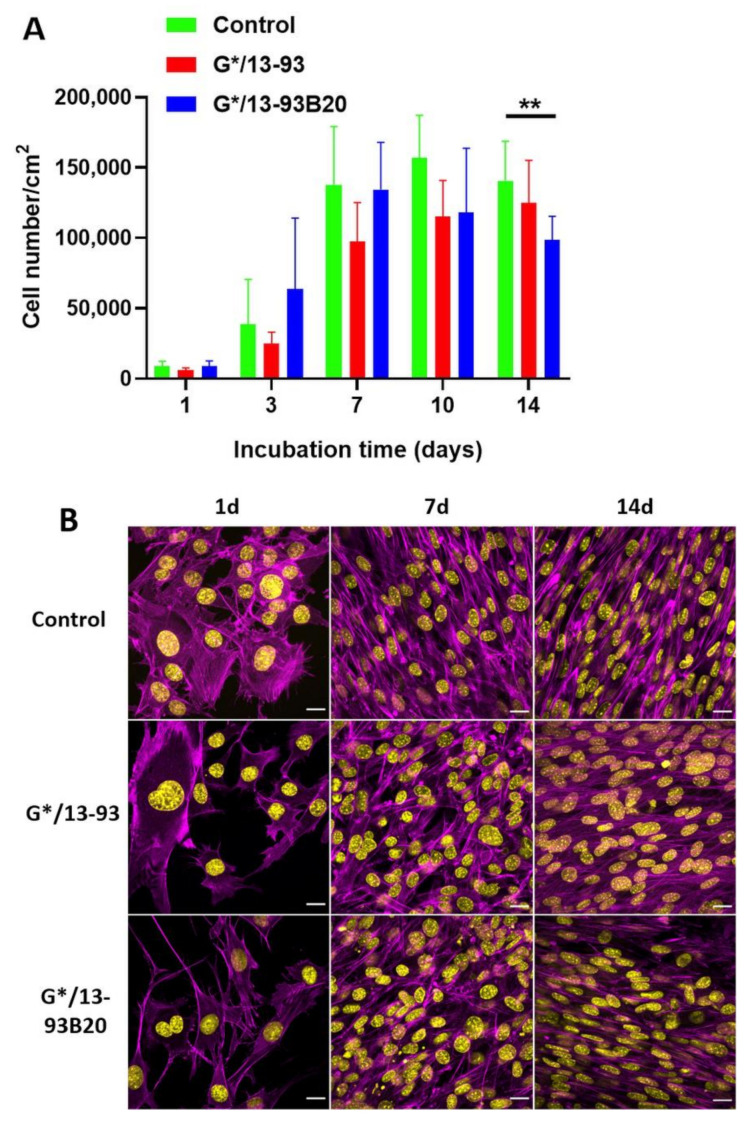
(**A**) Proliferation of MC3T3-E1 cells cultured in α-Minimum Essential Media (α-MEM) complete medium on Tissue Culture Polystyrene (TCPS), G*/13-93, and G*/13-93B20 hybrids for 14 days, analyzed by a CyQUANT Cell Proliferation Assay kit. The number of cells was normalized with the area of the materials surface (** *p* < 0.01). (**B**) Morphology of MC3T3-E1 cells in αMEM complete medium analyzed by nuclei (4’,6-Diamidino-2-phenylindole dihydrochloride (DAPI)—yellow) and actin (phalloidin—magenta) immunostaining after 1 day, 7 days, and 14 days of culture. Scale bar 20 µm.

MC3T3-E1 pre-osteoblastic cells were used to study their proliferation and morphology on the hybrids (Figure 10).

First, the number of MC3T3-E1 cells on the hybrids was studied for up to 14 days (Figure 10A). The hybrids were immersed 10 days in TRIS before cell culture to eliminate unreacted elements, which can prevent cell survival [38]. The hybrids were placed in 48 well plates, and the TCPS was used as a control. However, during their immersion in TRIS, they underwent a shrinking effect due syneresis. Indeed, they went from a diameter of 11 mm to 8 mm in 10 days. Thus, to compare the proliferation on the control and the materials, the cell number was normalized to the area of the respective sample.

For each condition, the cells proliferated with time and reached a plateau indicating the stationary phase (Figure 9A). The glass 13-93 alone was already known to promote cell adhesion and proliferation, as demonstrated by Fu et al. and Eqtesadi et al. [39,40]. At 14 days, the proliferation of MC3T3-E1 cells on G*/13-93B20 is significantly lower than on the control. This can be attributed to the release of boron from borosilicate glass, known to decrease cell proliferation while promoting osteogenesis, as observed in previous studies [13,24]. 

The morphology of the cells was observed at 24 h, 7 days, and 14 days on the control, G*/13-93, and G*/13-93B20 (Figure 10B). After 24 h, it can be observed that the cells spread on both hybrid types with their characteristic polygonal morphology. There is no difference noted in the cytoskeleton of cells between the conditions. At 7 days and 14 days, multicellular layers are observed, covering the hybrids. These results show that the cells can spread, attach, and proliferate on the hybrids. Thus, 13-93, 13-93B20, and GPTMS do not present cytotoxic effects and do not prevent the proliferation and adhesion of MC3T3-E1 cells on the hybrids. 

## 4. Conclusions

Hybrids made of gelatin and BAG particles (silicate 13-93 and borosilicate 13-93B20) covalently linked with GPTMS were synthesized using the sol-gel method. The process of synthesis was optimized in order to obtain a content of organic/inorganic matter close to that expected and to avoid particle sedimentation and aggregates. These hybrids were stable and self-supported at biological temperature in aqueous medium. When immersed in simulated body fluid, their bioactivity was shown. Cell survival was demonstrated using MC3T3-E1 cells. The substitution of 20% of SiO_2_ with B_2_O_3_ allowed us to tailor the dissolution and bioactivity properties of the hybrids. Once stabilized, the hybrids exhibited mechanical properties which, combined with their ability to precipitate HA and their biocompatible characteristic, make these materials good candidates for bone tissue engineering. Future studies will be conducted to investigate the osteo-stimulation of these materials.

## Figures and Tables

**Table 1:** Nominal glass composition (%).

Glass	mol%						
	Na_2_O	K_2_O	MgO	CaO	P_2_O_5_	SiO_2_	B_2_O_3_
13-93	6.0	7.9	7.7	22.1	1.7	54.6	-
13-93B20	6.0	7.9	7.7	22.1	1.7	43.7	10.9

**Table 2:** Measured glass loading and Young’s modulus of the gelatin alone functionalized (G*) and the G*/13-93 and G*/13-93B20 hybrids (for wet samples, the mechanical properties were measured after 10 min of immersion in TRIS).

Materials	Glass Loading in the Hybrids (wt%)	Young Modulus (MPa)	
		Dry Samples	Wet Samples
G* alone	**-**	2.1 ± 0.3	0.8 ± 0.2
G*/13-93	34 ± 2	0.5 ± 0.3	0.5 ± 0.1
G*/13-93B20	33 ± 1	0.7 ± 0.2	0.6 ± 0.2
